# Ozone treatment effectively eliminates SARS-CoV-2 from infected face masks

**DOI:** 10.1371/journal.pone.0271826

**Published:** 2022-07-22

**Authors:** Elizabeth Córdoba-Lanús, Omar García-Pérez, Francisco Rodríguez-Esparragón, Carlos J. Bethencourt-Estrella, Laura B. Torres-Mata, Angeles Blanco, Jesús Villar, Oscar Sanz, Juan J. Díaz, José L. Martín-Barrasa, Pedro Serrano-Aguilar, José-Enrique Piñero, Bernardino Clavo, Jacob Lorenzo-Morales

**Affiliations:** 1 Instituto Universitario de Enfermedades Tropicales y Salud Pública de Canarias de la Universidad de La Laguna, La Laguna, Tenerife, Spain; 2 Departamento de Medicina Interna Dermatología y Psiquiatría Universidad de La Laguna, La Laguna, Tenerife, Spain; 3 Red Cooperativa de Enfermedades Tropicales (RICET), Instituto de Salud Carlos III, Madrid, Spain; 4 CIBER de Enfermedades Infecciosas (CIBERINFEC), Instituto de Salud Carlos III, Madrid, Spain; 5 Research Unit Hospital Universitario Dr Negrín, Instituto de Investigación Sanitaria de Canarias (IISC), Las Palmas de Gran Canaria, Spain; 6 Fundación Canaria del Instituto de Investigación Sanitaria de Canarias (FIISC), Las Palmas de Gran Canaria Spain; 7 Departamento de Obstetricia, Ginecología, Pediatría, Medicina Preventiva y Salud Pública, Toxicología, Medicina Legal y Forense y Parasitología, Universidad de La Laguna La Laguna, Tenerife, Spain; 8 Instituto Universitario de Investigaciones Biomédicas y Sanitarias (IUIBS), BioPharm Group Universidad de Las Palmas de Gran Canaria, Spain; 9 Chemical Engineering & Materials Department, Universidad Complutense, Madrid, Spain; 10 CIBER de Enfermedades Respiratorias, Instituto de Salud Carlos III, Madrid, Spain; 11 Li Ka Shing Knowledge Institute at the St Michael’s Hospital, Toronto, Ontario, Canada; 12 Internal Medicine and Infectious Diseases Department, Hospital Universitario Dr Negrín, Instituto de Investigación Sanitaria de Canarias (IISC), Las Palmas de Gran Canaria, Spain; 13 Intensive Care Unit, Hospital Universitario Dr Negrín, Instituto de Investigación Sanitaria de Canarias (IISC), Las Palmas de Gran Canaria, Spain; 14 Animal Infectious Diseases and Ictiopathology, Universitary Institute of Animal Health and Food Safety (IUSA), Universidad de Las Palmas de Gran Canaria, Arucas, Spain; 15 RETIC de Investigación en Servicios de Salud en Enfermedades Crónicas (REDISSEC), Instituto de Salud Carlos III, Madrid, Spain; 16 Servicio de Evaluación y Planificación del Servicio Canario de Salud (SESCS), Santa Cruz de Tenerife, Spain; 17 Red de Agencias de Evaluación de Tecnologías Sanitarias y Prestaciones del Sistema Nacional de Salud (RedETS), Madrid, Spain; 18 Chronic Pain Unit Hospital Universitario Dr Negrín Las Palmas de Gran Canaria, Spain; 19 Radiation Oncology Department, Hospital Universitario Dr Negrín Las Palmas de Gran Canaria, Spain; Defense Threat Reduction Agency, UNITED STATES

## Abstract

The current COVID-19 pandemic is causing profound health, economic, and social problems worldwide. The global shortage of medical and personal protective equipment (PPE) in specialized centers during the outbreak demonstrated the need for efficient methods to disinfect and recycle them in times of emergency. We have previously described that high ozone concentrations destroyed viral RNA in an inactivated SARS-CoV-2 strain within a few minutes. However, the efficient ozone dosages for active SARS-CoV-2 are still unknown. The present study aimed to evaluate the systematic effects of ozone exposure on face masks from hospitalized patients infected with SARS-CoV-2. Face masks from COVID-19 patients were collected and treated with a clinical ozone generator at high ozone concentrations in small volumes for short periods. The study focused on SARS-CoV-2 gene detection (assessed by real-time quantitative polymerase chain reaction (RT-qPCR)) and on the virus inactivation by *in vitro* studies. We assessed the effects of different high ozone concentrations and exposure times on decontamination efficiency. We showed that high ozone concentrations (10,000, 2,000, and 4,000 ppm) and short exposure times (10, 10, and 2 minutes, respectively), inactivated both the original strain and the B.1.1.7 strain of SARS-CoV-2 from 24 contaminated face masks from COVID-19 patients. The validation results showed that the best condition for SARS-CoV-2 inactivation was a treatment of 4,000 ppm of ozone for 2 minutes. Further studies are in progress to advance the potential applications of these findings.

## Introduction

The current pandemic of coronavirus disease 2019 (COVID-19) that emerged in China in late December 2019 is caused by a novel human coronavirus named severe acute respiratory syndrome coronavirus 2 (SARS-CoV-2, first named HCoV-19) [1]. SARS-CoV-2 is spread primarily via respiratory droplets and aerosols during talking, coughing, or sneezing, but also fomites are a potential risk [2]. SARS-CoV-2 was reported to persist on different surfaces such as plastic, stainless steel, aluminum, wood, copper, and cardboard, at room temperature, showing stability for up to 4–5 days for a 10^4^ viral titer initial infection [3,4]. The survival time of the SARS-CoV-2 virus depends on environmental conditions, such as humidity, temperature, UV irradiation, or sun exposition [4–9].

SARS-CoV-2 persistence was documented on experimentally contaminated (titer of 7.88 LogTCID50/mL) personal protective equipment (PPE) materials (N-95 and N-100 masks; plastic visor shields) for up to 21 days [10]. Moreover, SARS-CoV-2 can remain viable for up to 2 weeks at room temperature on Tyvek 400 Coveralls (TY125SWH), a porous material [10]. In accordance, different studies were performed to test PPE decontamination by different methods such as heat, vaporous hydrogen peroxide, and UV light [11–13].

The need for an effective method of virus decontamination for emergencies, such as the global shortage of medical and PPE that hospitals, laboratories, and nursing centers suffered during the COVID-19 outbreak, was demonstrated [14]. The US Food and Drug Administration (FDA) issued Emergency Use Authorizations (EUAs) for certain PPE products, including face shields, other barriers, and respiratory protective devices such as respirators for decontamination procedures during the pandemic, revoking it in June 2021 (FDA 2021). Currently, there are no manufacturer-authorized methods for PPE decontamination. We have previously described that high ozone concentrations (above 4,000 ppm) on PPE could destroy RNA in a heat-inactivated SARS-CoV-2 strain within a few minutes [5].

The present study aimed to evaluate the systematic effects of ozone (O_3_) exposure on patients’ face masks naturally infected with SARS-CoV-2. We assessed different high ozone concentrations and exposure times to evaluate their potential for virus inactivation.

## Materials and methods

Between January and June of 2021, we evaluated the efficacy of viral inactivation by ozone treatment on FFP2 (KN 95) face masks infected with SARS-CoV-2 collected from patients with a COVID-19 diagnosis, hospitalized in the Infectious Diseases Department and the Intensive Care Unit at the Hospital Universitario de Gran Canaria Dr. Negrín (HUGCDN), Las Palmas de Gran Canaria, Spain. No other virus infection was determined for the studied patients. This study was approved by the Regional Ethics Committee (CEIm HUGCDN, Las Palmas, Spain, code #2020-183-1 COVID-19).

Ozone exposure experiments were performed at the HUGCDN. All genetic and molecular biology experiments were carried out at the Instituto Universitario de Enfermedades Tropicales y Salud Pública de Canarias (La Laguna, Tenerife, Spain) in a Biosafety Level 3 (BSL-3) laboratory. Briefly, face masks were collected 1 to 2 days after the onset of the patient’s admission. Once collected, face masks were folded in half to cut out a circular portion of a 3cm diameter that was then cut into 6 pieces of around 10×10 mm. Two pieces were used as a control (untreated) and 4 pieces (two in duplicate) were exposed to different ozone concentrations and exposure times. The samples were stored at room temperature until infection of VERO cells and subsequent genetic analysis were performed. Genomic analyses by quantitative real-time polymerase chain reaction (RT-qPCR) for the detection of 3 viral genes were performed in duplicate for each collected sample, before and after the *in vitro*-assays.

### Ozone exposure details

Accurate ozone concentrations were obtained using a portable medical ozone generator (Ozonobaric P®, Sedecal, Madrid, Spain), and medical grade O_2_. An O_3_/O_2_ gas mixture was obtained with O_3_ concentrations of 2,000, 4,000, and 10,000 ppm (4, 8, and 20 g/m^3^ respectively), to fill syringes with small volumes (60 mL syringes) at a standard room temperature (22.0–23.7°C) and relative humidity (53%-65%). The protocol used for ozone exposure was explained in detail previously [5].

Face mask samples were exposed to ozone in two phases: 1) an exploratory phase, which consecutively evaluated five different combinations of ozone concentrations and exposure times: 10,000 ppm for 10 min, 2,000 ppm for 10 min, 2,000 ppm for 5 min, 4,000 ppm for 1 min and 4,000 ppm for 2 min; and 2) a validation phase, for confirmatory evaluation of selected combinations of concentrations of ozone and times of exposure. In both phases, the management of face masks was similar. For each infected face mask, we obtained several samples (in duplicate): 1) a control sample without ozone treatment for RT-qPCR assessment, 2) a sample post-ozone treatment for RT-qPCR, and 3) a sample post-ozone treatment for *in vitro* assays. After cell exposure with mask samples, an additional RT-qPCR assay was performed in VERO cell lysates after 48hs.

### *In vitro* SARS-CoV-2 viral infection

Briefly, VERO E6 cells (ATCC CRL-1586) were cultured and maintained in DMEM (Dulbecco´s Modified Eagle Medium) supplemented with 10% fetal bovine serum (FBS) and 100U/mL of penicillin-streptomycin. Cells were cultured at 37°C and 5% CO_2_ (Gibco, Grand Island, NY, USA) in 6 and 24 well plates (Nunc, ThermoFisher, Madrid, Spain) until an almost confluent monolayer was formed (10^6^ cells/ml). To infect cultured cells with the virus, monolayers were washed twice with phosphate-buffered saline (PBS) and inoculated with SARS-CoV-2 from a naturally infected face mask sample kept in non-supplemented DMEM. Non-infected control cultures (mock/negative control) were prepared using non-supplemented DMEM as inoculum. Positive control of infection was carried out using the human coronavirus 229E ATCC ® VR-740 ™ strain (ATCC, LG Promochem, Barcelona, Spain). All the experiments were done in duplicate. Cell monolayers were checked daily under a Leica DM6000 inverted light microscope for the presence of cytopathic effects (CPE) for up to 48 h post-infection. The cell lysate was collected from wells by gentle scraping and pipetting, for further RT-qPCR assays.

### Real-time polymerase chain reaction (RT-qPCR)

RT-qPCR was used to detect viral RNA according to Spanish guidelines for biosafety level-2 facilities. RNA was extracted using the Maxwell 16S Viral RNA Mini Kit (Promega, Madrid, Spain) following the manufacturer´s recommendation. Briefly, each PPE infected sample was placed in 500 μL of inactivated medium (NEST Scientific, USA). Similarly, 200–300 μl of the cell lysates infected with SARS-CoV-2 from mask samples were analyzed by this method. After this step, 200–300 μL of the sample (mask samples in inactivated medium or cell lysates) was mixed with 300 μL of lysis buffer and 30 μL of proteinase K and vortexed for 20 seconds to proceed with the RNA extraction procedure (manufacturer instructions). The resulting RNA was eluted in 50 μL and conserved at -20°C until further use. For the amplification of the SARS-CoV-2 genes, the TaqPathTM 1-Step RT-qPCR Master Mix and TaqPath™ COVID-19 CE-IVD RT-qPCR Kit (Applied Biosystems Thermo Fisher Scientific, Madrid, Spain) were used in the RT-qPCR assays following the manufacturer´s instructions.

This Multiplex Assay allows the qualitative detection and characterization of SARS-CoV-2 RNA. Briefly, the kit included three assays that target SARS-CoV-2 genes (Genes ORF1ab, N Protein, S Protein), a control of RNA extraction the MS2 Phage Control, and a positive TaqPath™ COVID-19 Control. All the experiments were performed in duplicate in a QuantStudio5™ Real-Time qPCR System (Applied Biosystems). Positive results were considered when amplification genes had Ct values ≤37 (Ct: Cycle threshold related to the number of cycles required for the fluorescently marked amplification to cross the threshold in the RT-qPCR reaction). A lower cycle threshold value in the RT-qPCR indicates a higher viral load. SARS-CoV-2 B.1.1.7 dtec-RT-qPCR kit (Genetic PCR Solutions^TM^, Orihuela, Spain) that did not amplify the S gene was used for confirmation and detection of the UK-variant B.1.1.7 in those samples.

## Results

Six independent experiments were conducted to assess mask contamination by SARS-CoV-2. Thirty-six face masks were obtained from 36 COVID-19 hospitalized patients. We did not detect viral RNA in the five face masks obtained from patients admitted into the Intensive Care Unit. Twenty-four (67%) of the 31 assessed face masks from inpatients at the Infectious Disease Department were positive for SARS-CoV-2 genes and treated with ozone. Since February 2021, most COVID-19 patients were infected with the SARS-CoV-2 B.1.1.7 variant.

Mask samples were selected as appropriate for *in vitro* contamination experiments when showing median Ct values (24>Ct>30) assessed by RT-qPCR (positive control of the SARS-CoV-2 detection kit showed a median Ct value of 28 for the three genes). Seven masks were not evaluable for different technical reasons (S1 Table). Finally, 21 samples from 17 patients’ face masks were assessed for *in vitro* contamination of VERO cells. See details in Table 1 (The results of all the assessments performed in the study are shown in S1 and S2 Tables).

**Table 1:** Gene amplification by quantitative real-time polymerase chain reaction (RT-qPCR) in VERO cell lysates infected with FFP2 samples and treated at different ozone concentrations and exposure times.

Ozone Concentration/ time of exposure	Face masks from hospitalized COVID-19 patients						
**Preliminary Phase**							
***Assay 1***	**mask1**		**mask 2**		**mask 3**		**mask 4**
10,000 ppm 10 min	X		X		-		-
2,000 ppm 10 min	-		-		X		X
Control (Without O_3_ treatment)	√√√		√√√		√√√		√√√
***Assay 2***	**mask 6**	**mask 7**	**mask 8**	**mask 9**	**mask 12**	**mask 13**	**mask 15**
2,000 ppm 5 min	√^&^	X	X	-	-	X^†^	√^&^
4,000 ppm 1 min	-	-	-	X	X^†^	-	-
4,000 ppm 2 min	-	-	-	-	-	X	X
Control (Without O_3_ treatment)	√√√	√√√	√√√	√√√	√√√	√√*	√√*
**Validation Phase:**	**mask 17**	**mask 18**	**mask 19**	**mask 20**	**mask 23**	**mask 24**	
2,000 ppm 5 min	-	-	-	-	X	X	
4,000 ppm 2 min	X	X	X	X	X	X	
Control (Without O_3_ treatment)	√√*	*√√√*	√√*	*√√√*	√√*	√√*	

SARS-CoV-2 was detected by RT-qPCR. √: amplification of 1 virus-specific gene; √√: amplification of 2 virus genes (N, O); and √√√ 3 virus genes (N, S, O); X: negative detection of SARS-CoV-2; (-) not analysed under those conditions; * Masks positive for SARS-CoV-2 B117 variant; & VERO cell lysates from masks 6 and 15 showed amplification of 1 virus gene by RT-qPCR but not SARS-CoV-2 viability in the cultures; † In VERO cell lysates from masks 12 and 13 no gene amplification was detected but *in vitro* study showed partial cell lysis.

To elucidate whether the viral RNA resisted O_3_ treatment even if the virus itself is inactivated, RT-qPCR was performed using the masks as a template. RT-qPCR detection remained positive in all face mask samples after treatment with O_3_, showing amplification of 2 or 3 SARS-CoV-2 genes in all samples, except for 3 samples from 2 face masks (samples 13, 21, and 22) where only 1 gene was detected (S1 and S2 Tables).

**Fig 1:**
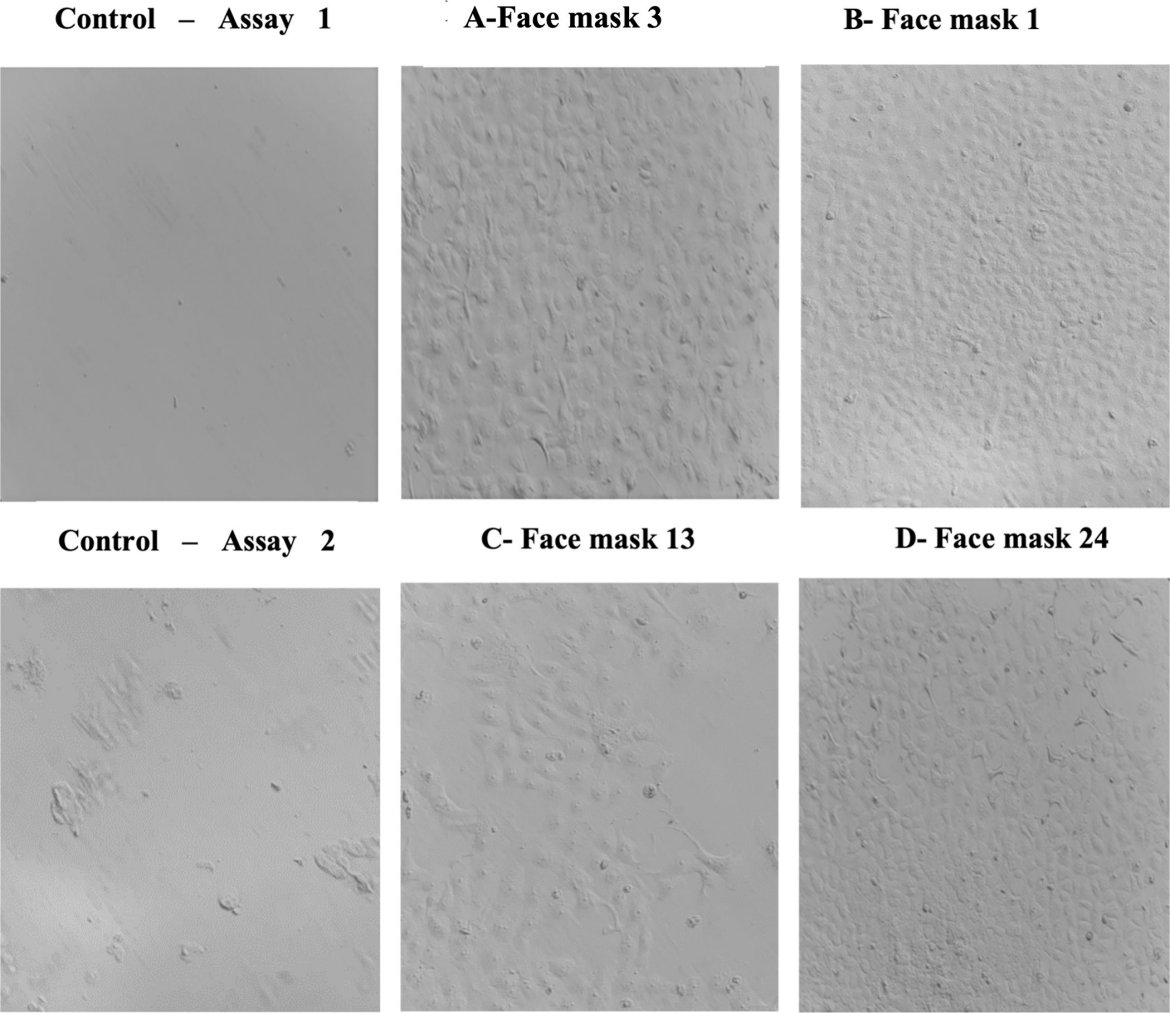
Images of VERO cells monolayer (x 20) after 48 h of incubation with patient´s face masks treated with different ozone concentrations and exposure times in the preliminary phase (assays 1 and 2). A and B show no cytopathic effect after cell culture incubation of face mask 3 treated with ozone at A- 2,000 ppm for 10 min and face mask 1 treated at B- 10,000 ppm for 10 min. Image C- shows the partial lysis of the cell monolayer observed when incubation with face mask 13 treated at ozone concentrations of 2,000 ppm for 5 min. Image D- shows the effect of exposure to face mask 24 treated at 4,000 ppm for 2 min where cells are alive suggesting no viability of SARS-CoV-2 under this condition. The positive control consisted of VERO cells infected with human coronavirus 229E ATCC ® VR-740 ™ strain. Images are representative of experiments performed in triplicate.

When face mask samples contaminated with SARS-CoV-2 without ozone treatment were used to infect VERO cells in *in vitro* experiments, an immediate cytopathic effect of monolayer cells was observed after 48 h of incubation. However, in the preliminary phase, a significant increase in the percentage of VERO cells survival was observed after higher O_3_ concentration or time-exposition. There were no viral effects on VERO cells using face mask samples treated with O_3_ at 10,000 for 10 min, 2,000 for 10 min, or 4,000 ppm for 2 min. Cells incubated under standard conditions were used as negative controls of the cytopathic effect and showed no alteration during the experiments (Fig 1). Partial lysis was observed in VERO cell culture at 2,000 ppm for 5 min and 4,000 ppm for 1 min. Only one gene was detected by RT-qPCR in VERO cell lysates from face masks 6 and 15 (assay 2) treated with O_3_ at 2,000 ppm for 5 min, although the virus was not viable *in vitro*.

**Fig 2:**
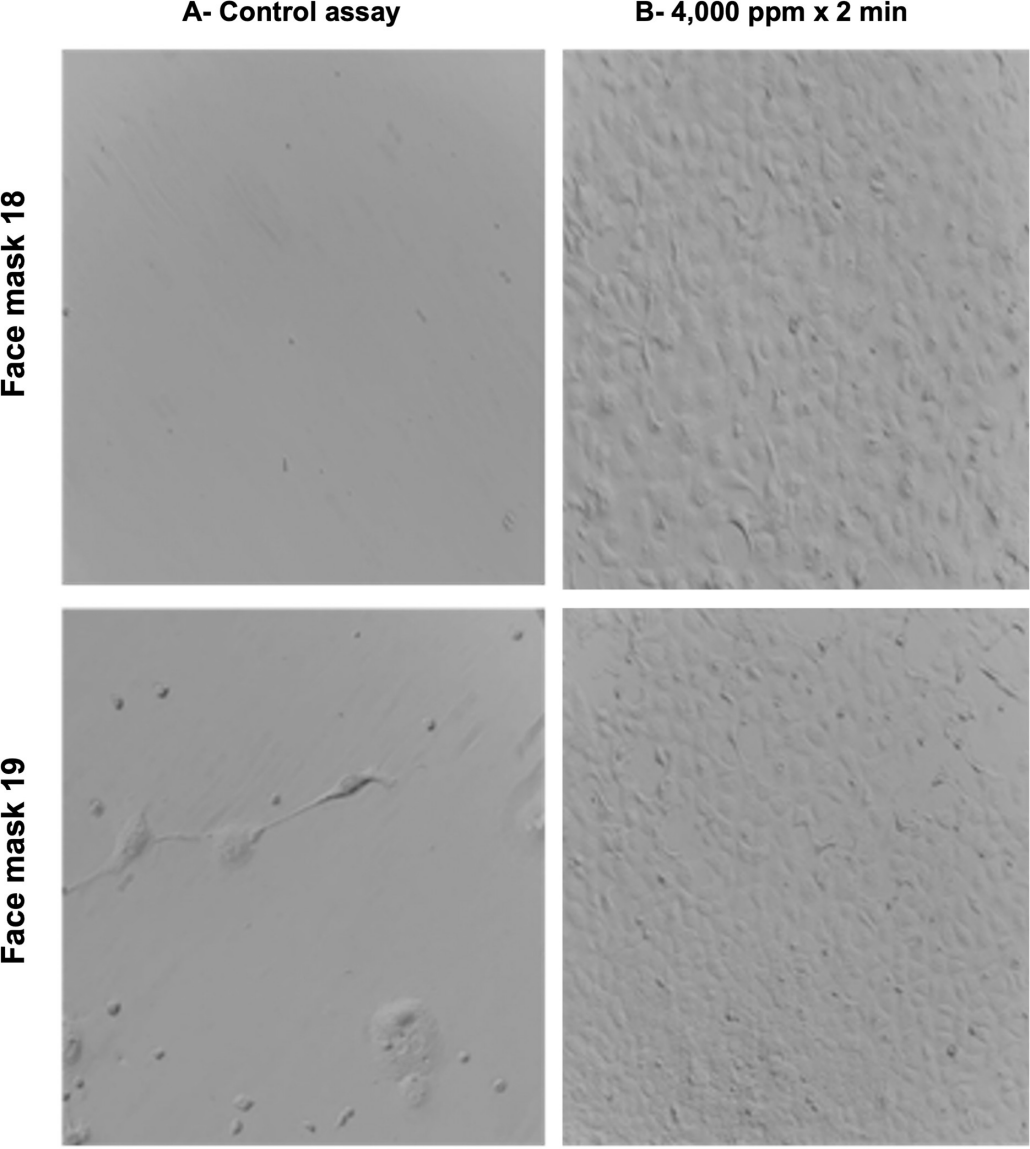
Representative images of VERO cells monolayer (x 20) after 48 h of incubation of VERO cell culture of face masks 18 and 19 from patients analyzed in the validation phase. Left: The positive control consisted of VERO cells infected with human coronavirus 229E ATCC ® VR-740 ™ strain, which were completely lysed. Right: face masks 18 (upper) and 19 (lower) that were treated with ozone at 4,000 ppm for 2 min, showed no cytopathic effect (no viability of SARS-CoV-2). Experiments were performed in triplicate.

Viral RNA was detected in the lysates from VERO cells infected with face mask samples without O_3_ treatment and used as a positive control. There was no viral gene detection by RT-qPCR in all the other culture lysates collected from cells exposed to face mask samples treated with O_3_ at 10,000 ppm for 10 min, 2,000 ppm for 10 min, and 4,000 ppm for 2 min in the preliminary and validation phase (Fig 2) (Tables 1 and S1).

## Discussion

This study shows the potential utility of ozone treatment in the inactivation of SARS-CoV-2 in face masks collected from COVID-19 patients. Ozone treatment voids SARS-CoV-2 replication in all contaminated face masks treated at 10,000 ppm for 10 min, 2,000 ppm for 10 min, and 4,000 ppm for 2 min. However, the effect with shorter exposure times did not show conclusive results (2,000 ppm for 5 min, and 4,000 ppm for 1 min). Thus, ozone treatment can inactivate SARS-CoV-2, but an appropriate combination of ozone concentration and exposure time is the main determinant of efficacy.

In the first steps of the exploratory phase, we evaluated the viricidal effect of higher combinations of ozone concentrations of 10,000 ppm (20–4 g/m^3^) and exposure time (10 min). The selection was based on our previous study [5]. In the current study, we use naturally infected SARS-CoV-2 samples instead of a heat-inactivated SARS-CoV-2 strain which led us to choose a “concentration-time” range slightly higher than in previous experiments [5]. Based on the results obtained from the exploratory phase, we determined the optimized ozone exposure concentrations and times. In the second step, we evaluated the ozone effect on SARS-CoV-2, with three combinations of ozone concentration and time exposure: 4,000 ppm for 2 min and 1 min, and 2,000 ppm for 5 min, resulting in 4,000 ppm for 2 min the best combination, which was validated in the second phase. It is worth mentioning that the ozone “concentration and time of exposure” conditions used in this study to inactivate natural SARS-CoV-2 in face masks are the same as we described for viral RNA elimination using a heat-inactivated SARS-CoV-2 strain in our previous work. This fact could be of potential relevance for the planning of further studies.

SARS-CoV-2 has been reported to persist on different surfaces for several days [3,4]. Moreover, previous *in vitro* assay data from our group support that face masks infected with SARS-CoV-2 maintained their infectiveness viability for 5 to 7 days after contamination [15]. Ozone impacts different targets in viruses, including the viral capsid, specific viral attachment epitopes into new cell hosts, or damaging viral RNA [16]. In agreement with this data, our study showed that viral genetic material can still be detected (by RT-qPCR) although the virus may be efficiently inactivated by ozone (*in vitro* assay). Therefore, ozone concentration and time of exposure for the inactivation of SARS-CoV-2 to void the capacity of replication and infectivity is lower than is required for complete virus destruction (including nucleic acids). This data suggests that in SARS-CoV-2, the peroxidation of lipids of the capsid and viral envelope occurs before RNA damage, as it has been described for herpes simplex virus and adenovirus [17]. These also agree with the reported damage produced by ozone in the virus protein structures and viral envelope [18]. On the other hand, it has been reported that RNA damage is the principal mechanism for viral inactivation in poliovirus and echovirus [16,19,20]. It is relevant to keep this differential effect in mind when comparing studies between natural SARS-CoV-2 and heat-inactivated viruses, and it could have practical implications.

In previous research from our group [15] we observed that due to its porous composition, FPP2 face masks seem to be more suitable for virus stability when compared to other materials, as has already been reported previously for other viruses [21]. So, it is important to implement a reliable method with a viricidal effect to treat PPE material. It was suggested that ozone, as a gas, could penetrate different textures and fabric compositions. It is relevant to consider the use of many certified PPE materials of varied fabric compositions and the diverse effects of ozone on SARS-CoV-2 according to the different materials.

Ozone gas`efficiency in disinfection against SARS-CoV-2 is still under study [7]. The efficiency of ozone depends on the total dosage, ozone concentration, exposure time, temperature, and relative humidity in the environmental conditions [4–8], even sunlight exposure [9]. High ozone concentrations are adequate for achieving SARS-CoV-2 inactivation in a few minutes, as we were able to verify in our study. Indeed, according to this study and our previous one [5], this effect could probably be obtained in lower exposure time (half a minute with even higher concentrations of 10,000–40,000 ppm (20–40 g/m^3^ respectively) by medical ozone generators. This fact could open the possibility for treatment of full PPE, as we suggested (Comment #224) in answer to a “Call for Ideas for Conserving Supply of PPE” by the editors of the Journal of the American Medical Association (JAMA, 2020) [22]. However, high concentrations of ozone are not easily obtainable in large volumes in a short time. Ozone inhalation due to exposure to high concentrations is potentially harmful and should be reduced as much as possible. Further research about potential combinations of ozone concentration, volume, and time of exposition is required.

Our study evaluated ozone concentrations at 2,000, 4,000, and 10,000 ppm (4, 8, and 20 g/m^3^, respectively), which are higher doses than those evaluated in other studies [7]. A review of experimental studies in surface disinfection of several viruses showed a 99.99% viral reduction for ozone concentrations from 65 ppm to 15 ppm (13–30 mg/m^3^), but for longer periods of 120–50 min [7]. One study evaluated disinfection of the human coronavirus HCoV-229E in face masks using ozone produced by a dielectric barrier discharge plasma generator, reporting a 4-log viral reduction after ozone exposures at 120 ppm (0.24 g/m^3^) during 10 s, however, contamination was done by spraying a 250 μL of a virus solution of 45 logs TCID50/mL [23]. Additionally, it has been reported that the ozone concentration required for effective airborne control of norovirus (also an RNA virus from Group IV) is very low [24], as is the inactivation of SARS-CoV-2 *in vitro* [25]. However, conditions in our study were very different: first, the inner side of face masks was in contact with the mouth and nose of COVID-19 patients for 6 to 8 hours, which should result in a much higher SARS-CoV-2 concentration [26]; secondly, virus theoretically was present throughout the thickness of the porous face mask, and this condition would need higher doses than those required to inactivate easily accessible airborne, monolayer culture, or surface contamination by SARS-CoV-2; thirdly, patients’ face masks may contain respiratory mucous secretions and biological compounds with antioxidant activity.

This study aimed to verify the role of ozone in inactivating SARS-CoV-2 from face masks. Low concentrations of ozone, as low as 0.23 ppm for 40 min, have been reported to be effective in controlling other airborne viruses [24]. This effect is additionally supported by the described inverse correlation between environmental low O_3_ levels (from 0.049 to 0.095 mg/m^3^ = from 0.025 to 0.048 ppm) and SARS-CoV-2 transmission [27]. However, using low O_3_ concentrations within the OSHA (Occupational Safety Health regulations) exposure limits (0.1 ppm, 0.20 mg/m^3^, for 8 hours of O_3_ exposition) [28], a much larger exposure time is required, and further research in this field is needed.

We acknowledge some limitations to our study. First, 67% of face masks obtained from COVID-19 patients did not show positive detection for viral RNA by qPCR. Indeed, no face masks from Intensive Care Unit patients showed SARS-CoV-2, probably because they were patients with a long hospital stay. However, this finding agrees with the 60% described by other authors [26]. Second, we cannot discount the possibility that ozone effects could potentially differ in different strains, although our study showed similar effects in both the original and B117 strains of SARS-CoV-2. Third, patients´ face masks were randomly selected and the viral load present in them was not quantified. However, to minimize this effect, face masks that resulted in positive detection of SARS-CoV-2 genes within 20>Ct>30 values were included for the *in vitro* analysis. In our study, the inner side of evaluated face masks was in close contact with the mouth and nose of COVID-19 inpatients while breathing for several hours. Some reports support this fact as important since a different risk was observed between the inner and outer sides [26]. Fourth, since we used 60 mL syringes to dispense the ozone, we used conventional room temperature and relative humidity at Hospital facilities. However, optimization could be possible by modifying these factors using ozonation chambers, as suggested by our previous study and by other authors [4–9].

Further studies are needed to validate the present findings in different PPE materials, although the potential benefits are worth the effort.

## Conclusions

SARS-CoV-2 in face masks from COVID-19 hospitalized patients can be inactivated by high ozone concentrations within a few minutes. The best-evaluated option in this study was 4,000 ppm (8 g/m^3^) 2 min, although shorter exposure times could be required for higher concentrations. Appropriate ozone application in concentration and time exposure could be useful for SARS-CoV-2 inactivation in face masks and PPE. This could be useful for decreasing the risk associated with the management of PPE and, potentially, other medical and general equipment. Further research is in progress.

## Supporting information

S1 Table Face mask samples were treated with ozone at different concentrations and times of exposure. SARS-CoV-2 gene detection by RT-qPCR and *in vitro* assessment. RT-qPCR: quantitative real-time polymerase chain reaction. √: amplification by RT-qPCR of one SARS-CoV-2 gene. √√: amplification by RT-qPCR of two SARS-CoV-2 genes (N, O). √√√: amplification by RT-qPCR of 3 SARS-CoV-2 genes (N, S, O). X: negative detection of SARS-CoV-2 genes. XX: non-viability of SARS-CoV-2 in VERO cells culture.; X partial: partial lysis of infected cells. NP: non-processed sample. ^a^ Low viral load (Ct>30). ^b^ Very high viral load (Ct<20). * Face masks positive for SARS-CoV-2 B.1.1.7 variant. Every mask sample was analyzed in duplicate.
(DOCX)

S2 Table Face mask samples were treated with ozone at different concentrations and times of exposure. SARS-CoV-2 gene detection by RT-qPCR before and after *in vitro* assessment. RT-qPCR: quantitative real-time polymerase chain reaction. √: amplification by RT-qPCR of one SARS-CoV-2 gene. √√: amplification by RT-qPCR of two SARS-CoV-2 genes (N, O). √√√: amplification by RT-qPCR of 3 SARS-CoV-2 genes (N, S, O). na: no gene amplification. X: negative detection of SARS-CoV-2 genes. XX: non-viability of SARS-CoV-2 in VERO cells culture.; X partial: partial lysis of infected cells. NP: non-processed sample. ^a^ Low viral load (Ct>30). ^b^ Very high viral load (Ct<20). * Face masks positive for SARS-CoV-2 B.1.1.7 variant. Every mask sample was analyzed in duplicate.
(DOCX)

S1 Dataset (XLSX)

## Abbreviations

BSL-3: Biosafety Level 3
COVID-19: Coronavirus disease 2019
DMEM: Dulbecco´s Modified Eagle Medium
EUA: Emergency Use Authorizations
FBS: Fetal bovine serum
FDA: US Food and Drug Administration
HUGCDN: Hospital Universitario de Gran Canaria Dr Negrín
JAMA: Journal of the American Medical Association
OSHA: Occupational Safety and Health Administration
PBS: Phosphate buffered saline
PPE: Personal protective equipment
RT-qPCR: Real-time polymerase chain reaction
SARS-CoV-2: Severe acute respiratory syndrome coronavirus 2
VERO: African green monkey kidney epithelial cells
