# Supplementary material for: Ozone treatment effectively eliminates SARS-CoV-2 from infected face masks
Source: PLoS One. 2022 Jul 22;17(7):e0271826. doi: 10.1371/journal.pone.0271826 (PMC9307172; doi:10.1371/journal.pone.0271826)
Supplement: S1 Table — SARS-CoV-2 gene detection by RT-qPCR and in vitro assessment. RT-qPCR: quantitative real-time polymerase chain reaction. √: amplification by RT-qPCR of one SARS-CoV-2 gene. √√: amplification by RT-qPCR of two SARS-CoV-2 genes (N, O). √√√: amplification by RT-qPCR of 3 SARS-CoV-2 genes (N, S, O). X: negative detection of SARS-CoV-2 genes. XX: non-viability of SARS-CoV-2 in VERO cells culture.; X partial: partial lysis of infected cells. NP: non-processed sample. a Low viral load (Ct>30). b Very high viral load (Ct<20). * Face masks positive for SARS-CoV-2 B.1.1.7 variant. Every mask sample was analyzed in duplicate. (DOCX) [file pone.0271826.s001.docx]

**Table S1.** **Face mask samples were treated with ozone at different concentrations and times of exposure. SARS-CoV-2 gene detection by RT-qPCR and *in vitro* assessment.**

|  |  | **Ozone concentrations (ppm)/**  **exposure times (min)** | | | | | **RT-qPCR**  **after-O_3_** | | ***in vitro* assessment** |
| --- | --- | --- | --- | --- | --- | --- | --- | --- | --- |
| **Nº** | **COVID-19 patients`**  **face masks** | **10,000/**  **10 min** | **2,000/**  **10 min** | **2,000/**  **5 min** | **4,000 /**  **1 min** | **4,000 /**  **2 min** | **Masks**  **before**  ***in vitro***  **infection** | **Cells lysate**  **infected** | **Viability of SARS-CoV-2**  **in VERO cells** |
| **Preliminary Phase** | | | | | | | | | |
| **Assay 1** | | | | | | | | | |
| **1** | **mask 1** | • |  |  |  |  | √√√ | X | XX |
| **2** | **mask 2** | • |  |  |  |  | √√√ | X | XX |
| **3** | **mask 3** |  | • |  |  |  | √√√ | X | XX |
| **4** | **mask 4** |  | • |  |  |  | √√√ | X | XX |
| **Assay 2** | | | | | | | | | |
| **5** | **mask 5 ^a^** |  |  | • |  |  | NP | NP | NP |
| **6** | **mask 6** |  |  | • |  |  | √√√ | √ | XX |
| **7** | **mask 7** |  |  | • |  |  | √√√ | X | XX |
| **8** | **mask 8** |  |  | • |  |  | √√√ | X | XX |
| **9** | **mask 9** |  |  |  | • |  | √√√ | X | X X |
| **10** | **mask 10 ^b^** |  |  |  | • |  | NP | NP | NP |
| **11** | **mask 11 ^b^** |  |  |  | • |  | NP | NP | NP |
| **12** | **mask 12** |  |  |  | • |  | √√√ | X | X partial |
| **13** | ***mask 13** |  |  | • |  |  | √ | X | X partial |
| **14** | ***mask 13** |  |  |  |  | • | √ | X | XX |
| **15** | ***mask 14 ^b^** |  |  | • |  |  | √√ | NP | NP |
| **16** | ***mask 14 ^b^** |  |  |  |  | • | √√ | NP | NP |
| **17** | ***mask 15** |  |  | • |  |  | √√ | √ | XX |
| **18** | ***mask 15** |  |  |  |  | • | √√ | X | XX |
| **19** | ***mask 16 ^a^** |  |  | • |  |  | √√ | NP | NP |
| **20** | ***mask 16 ^a^** |  |  |  |  | • | √√ | NP | NP |
| **Validation** | | | | | | | | | |
| **21** | ***mask 17** |  |  |  |  | • | √√ | X | XX |
| **22** | **mask 18** |  |  |  |  | • | √√ | X | XX |
| **23** | ***mask 19** |  |  |  |  | • | √√ | X | XX |
| **24** | **mask 20** |  |  |  |  | • | √√√ | X | XX |
| **25*** | ***mask 21 ^a^** |  |  | • |  |  | √ | NP | NP |
| **26*** | ***mask 21 ^a^** |  |  |  |  | • | √ | NP | NP |
| **27** | ***mask 22 ^a^** |  |  | • |  |  | √ | NP | NP |
| **28** | ***mask 22 ^a^** |  |  |  |  | • | √ | NP | NP |
| **29** | ***mask 23** |  |  | • |  |  | √√ | X | XX |
| **30** | ***mask 23** |  |  |  |  | • | √√ | X | XX |
| **31** | ***mask 24** |  |  | • |  |  | √ | X | XX |
| **32** | ***mask 24** |  |  |  |  | • | √√ | X | XX |

RT-qPCR: quantitative real-time polymerase chain reaction. √: amplification by RT-qPCR of one SARS-CoV-2 gene. √√: amplification by RT-qPCR of two SARS-CoV-2 genes (N, O). √√√: amplification by RT-qPCR of 3 SARS-CoV-2 genes (N, S, O). X: negative detection of SARS-CoV-2 genes. XX: non-viability of SARS-CoV-2 in VERO cells culture.; X partial: partial lysis of infected cells. NP: non-processed sample. ^a^ Low viral load (Ct>30). ^b^ Very high viral load (Ct<20). * Face masks positive for SARS-CoV-2 B.1.1.7 variant. Every mask sample was analyzed in duplicate.
