# Supplementary material for: Ozone treatment effectively eliminates SARS-CoV-2 from infected face masks
Source: PLoS One. 2022 Jul 22;17(7):e0271826. doi: 10.1371/journal.pone.0271826 (PMC9307172; doi:10.1371/journal.pone.0271826)
Supplement: S2 Table — SARS-CoV-2 gene detection by RT-qPCR before and after in vitro assessment. RT-qPCR: quantitative real-time polymerase chain reaction. √: amplification by RT-qPCR of one SARS-CoV-2 gene. √√: amplification by RT-qPCR of two SARS-CoV-2 genes (N, O). √√√: amplification by RT-qPCR of 3 SARS-CoV-2 genes (N, S, O). na: no gene amplification. X: negative detection of SARS-CoV-2 genes. XX: non-viability of SARS-CoV-2 in VERO cells culture.; X partial: partial lysis of infected cells. NP: non-processed sample. a Low viral load (Ct>30). b Very high viral load (Ct<20). * Face masks positive for SARS-CoV-2 B.1.1.7 variant. Every mask sample was analyzed in duplicate. (DOCX) [file pone.0271826.s002.docx]

**S2 Table.** **Face mask samples were treated with ozone at different concentrations and times of exposure. SARS-CoV-2 gene detection by RT-qPCR before and after *in vitro* assessment.**

|  |  | **RT-qPCR before *in vitro* infection (median Ct value)** | | | | **RT-qPCR**  **after *in vitro assay*** | ***In vitro* assessment** |
| --- | --- | --- | --- | --- | --- | --- | --- |
| **Nº** | **COVID-19 patients`**  **face masks** | **Total**  **gene**  **amplification** | **Gene**  **S** | **Gene**  **O** | **Gene**  **N** | **Total gene amplification** | **Viability of SARS-CoV-2**  **in VERO cells** |
| **Preliminary Phase** | | | | | | | |
| **Assay 1** | | | | | | | |
| **10,000/10 min** | | | | | | | |
| **1** | **mask 1** | √√√ | 28 | 29 | 28 | X | XX |
| **2** | **mask 2** | √√√ | 27 | 28 | 29 | X | XX |
| **2,000/ 10 min** | | | | | | | |
| **3** | **mask 3** | √√√ | 27 | 26 | 26 | X | XX |
| **4** | **mask 4** | √√√ | 27 | 27 | 26 | X | XX |
| **Assay 2** | | | | | | | |
| **2,000/ 5 min** | | | | | | | |
| **5** | **mask 5 ^a^** | NP | 32 | 31 | 32 | NP | NP |
| **6** | **mask 6** | √√√ | 28 | 28 | 29 | √ | XX |
| **7** | **mask 7** | √√√ | 28 | 29 | 29 | X | XX |
| **8** | **mask 8** | √√√ | 25 | 26 | 27 | X | XX |
| **4,000 /1 min** | | | | | | | |
| **9** | **mask 9** | √√√ | 28 | 28 | 28 | X | XX |
| **10** | **mask 10 ^b^** | NP | 18 | 19 | 19 | NP | NP |
| **11** | **mask 11 ^b^** | NP | 19 | 20 | 20 | NP | NP |
| **12** | **mask 12** | √√√ | 28 | 29 | 29 | X | X partial |
| **2,000/ 5 min** | | | | | | | |
| **13** | ***mask 13** | √ | na | na | 29 | X | X partial |
| **14** | ***mask 14 ^b^** | √√ | na | 20 | 21 | NP | NP |
| **15** | ***mask 15** | √√ | na | 28 | 28 | √ | XX |
| **16** | ***mask 16 ^a^** | √√ | na | 34 | 34 | NP | NP |
| **4,000 /2 min** | |  |  |  |  |  |  |
| **17** | ***mask 13** | √ | na | 24 | 25 | X | XX |
| **18** | ***mask 14 ^b^** | √√ | na | 21 | 22 | NP | NP |
| **19** | ***mask 15** | √√ | na | 28 | 27 | X | XX |
| **20** | ***mask 16 ^a^** | √√ | na | 33 | 32 | NP | NP |
| **Validation Phase** | | | | | | | |
| **4,000 /2 min** | | | | | | | |
| **21** | ***mask 17** | √√ | na | 24 | 25 | X | XX |
| **22** | **mask 18** | √√√ | 29 | 28 | 28 | X | XX |
| **23** | ***mask 19** | √√ | na | 27 | 26 | X | XX |
| **24** | **mask 20** | √√√ | 28 | 29 | 28 | X | XX |
| **25** | ***mask 21 ^a^** | √ | na | na | 33 | NP | NP |
| **26** | ***mask 22 ^a^** | √ | na | na | 32 | NP | NP |
| **27** | ***mask 23** | √√ | na | 28 | 27 | X | XX |
| **28** | ***mask 24** | √√ | na | 28 | 28 | X | XX |
| **2,000/ 5 min** | | | | | | | |
| **29** | ***mask 21 ^a^** | √ | na | na | 32 | NP | NP |
| **30** | ***mask 22 ^a^** | √ | na | na | 34 | NP | NP |
| **31** | ***mask 23** | √√ | na | 28 | 29 | X | XX |
| **32** | ***mask 24** | √√ | na | na | 29 | X | XX |

RT-qPCR: quantitative real-time polymerase chain reaction. √: amplification by RT-qPCR of one SARS-CoV-2 gene. √√: amplification by RT-qPCR of two SARS-CoV-2 genes (N, O). √√√: amplification by RT-qPCR of 3 SARS-CoV-2 genes (N, S, O). na: no gene amplification. X: negative detection of SARS-CoV-2 genes. XX: non-viability of SARS-CoV-2 in VERO cells culture.; X partial: partial lysis of infected cells. NP: non-processed sample. ^a^ Low viral load (Ct>30). ^b^ Very high viral load (Ct<20). * Face masks positive for SARS-CoV-2 B.1.1.7 variant. Every mask sample was analyzed in duplicate.
